# Lymphoadenopathy during Lyme Borreliosis Is Caused by Spirochete Migration-Induced Specific B Cell Activation

**DOI:** 10.1371/journal.ppat.1002066

**Published:** 2011-05-26

**Authors:** Stefan S. Tunev, Christine J. Hastey, Emir Hodzic, Sunlian Feng, Stephen W. Barthold, Nicole Baumgarth

**Affiliations:** 1 Center for Comparative Medicine, University of California Davis, Davis, California, United States of America; 2 Graduate Group in Comparative Pathology, University of California Davis, Davis, California, United States of America; 3 Department of Pathology, Microbiology and Immunology, University of California Davis, Davis, California, United States of America; 4 Graduate Group in Microbiology, University of California Davis, Davis, California, United States of America; Medical College of Wisconsin, United States of America

## Abstract

Lymphadenopathy is a hallmark of acute infection with *Borrelia burgdorferi*, a tick-borne spirochete and causative agent of Lyme borreliosis, but the underlying causes and the functional consequences of this lymph node enlargement have not been revealed. The present study demonstrates that extracellular, live spirochetes accumulate in the cortical areas of lymph nodes following infection of mice with either host-adapted, or tick-borne *B. burgdorferi* and that they, but not inactivated spirochetes, drive the lymphadenopathy. The ensuing lymph node response is characterized by strong, rapid extrafollicular B cell proliferation and differentiation to plasma cells, as assessed by immunohistochemistry, flow cytometry and ELISPOT analysis, while germinal center reactions were not consistently observed. The extrafollicular nature of this B cell response and its strongly IgM-skewed isotype profile bear the hallmarks of a T-independent response. The induced B cell response does appear, however, to be largely antigen-specific. Use of a cocktail of recombinant, *in vivo*-expressed *B. burgdorferi*-antigens revealed the robust induction of borrelia-specific antibody-secreting cells by ELISPOT. Furthermore, nearly a quarter of hybridomas generated from regional lymph nodes during acute infection showed reactivity against a small number of recombinant Borrelia-antigens. Finally, neither the quality nor the magnitude of the B cell responses was altered in mice lacking the Toll-like receptor adaptor molecule MyD88. Together, these findings suggest a novel evasion strategy for *B. burgdorferi*: subversion of the quality of a strongly induced, potentially protective borrelia-specific antibody response via *B. burdorferi*'s accumulation in lymph nodes.

## Author Summary

Acute Lyme Disease is one of the most important emerging diseases in the US. People with acute Lyme disease often develop swollen lymph nodes, or lymphadenopathy, but we do not know why this happens or what effect it has on the course of the disease. We show here that when mice are infected with live *Borrelia burgdorferi* spirochetes (the bacteria that cause Lyme disease), live spirochetes collect in the lymph nodes. These lymph nodes then swell up and start producing large numbers of antibody-producing cells. Although many of these antibodies can recognize the bacteria, they apparently lack the quality to clear the infection. We hypothesize that by moving into the lymph node, usually a site in which strong immune responses are induced, Borrelia evades the immune response: it goes to the lymph nodes and tricks the immune system into making a very strong but inadequate response.

## Introduction

Lyme borreliosis, caused by *Borrelia burgdorferi* transmitted by *Ixodes spp.* ticks, is the most common arthropod-borne illness in the US and Europe, and is increasing in prevalence and expanding in geographic distribution in the US [1], [2]. Clinical manifestations are highly varied, including involvement of the cutaneous, cardiovascular, musculoskeletal, and nervous systems [3]–[5]. A frequent, but largely under-studied manifestation is massive and systemic lymph node enlargement (lymphadenopathy), observed particularly in the regional lymph node near the site of infection in humans, and in experimentally-infected dogs [4], [6]. The lymph node enlargement that arises in both humans and dogs is characterized by increased cellularity and the accumulation of large pleomorphic IgM- and IgG-positive plasma cells [6]–[8]. Despite these unusual characteristics, the lymphadenopathy of Lyme borreliosis has not been well investigated.

Several *in vitro* studies have shown that culture-grown *B. burgdorferi* can act as mitogens when co-cultured with human or murine naive B cells [9]–[16]. Therefore, the unusual lymphadenopathy of Lyme borreliosis might be a manifestation of non-specific B cell activation. Massive lymph node enlargement has also been seen in wildtype but not TLR4 gene-targeted mice during infection with *Salmonella typhimurium* [17] and others have shown a role for TLR-independent, TNF-independent [18] or TNF-dependent [19] involvement of mast cells in non-specific induction of lymph node enlargement. Thus, innate immune activation might account for the lymphadenopathy observed during infection with *B. burgdorferi*.

On the other hand, there is ample evidence for the induction of specific immune responses following *B. burgdorferi* infection. Both following experimental and natural infections, *B. burgdorferi*-specific IgM and IgG antibodies are induced in the serum of infected humans [5], [20]–[24], dogs [25], and mice [26], among other host species. Importantly, passive transfer of immune-serum from chronically infected wildtype or T cell-deficient mice, from naturally infected dogs, and from human patients with chronic Lyme disease can protect mice from a challenge infection with *B. burgdorferi* [26]–[29], demonstrating that specific and protective antibodies are induced during the course of infection. However, once infection is established, the immune response is incapable of clearing infection [26], [30]. Thus, understanding the host immune response is critical to understanding and treating Lyme borreliosis.

The present study was undertaken to identify the mechanisms involved in the lymphadenopathy induced by infection with *B. burgdorferi* and to determine the nature and specificity of the reactive B cell response. Using a mouse model of infection with host-adapted spirochetes that faithfully recapitulates experimental and natural infections with ticks, we show that *B. burgdorferi* actively migrates into the lymph nodes, where it causes a largely specific, but unusual B cell response.

## Materials and Methods

### Mice and infections

Four to six week old female C3H/He, C57BL/6 and severe combined immunodeficient C57BL/B6.C-*Prkdc^scid^* (SCID) mice were obtained from The Jackson Laboratory, Bar Harbor, ME, and maintained at UC Davis in isolator cages under conventional housing conditions. Breeding pairs of C57BL/6.129P2/Ola-*MyD88^tm1Aki^* (MyD88 −/−) mice [31] were a generous gift of Richard Flavell (Yale University), given with kind permission from Shizuo Akira (Osaka University). The MyD88−/− mice were rederived and bred in the specific pathogen free barrier facility at UC Davis, and then transferred to conventional housing prior to experiment onset.

Mice were infected with *B. burgdorferi* in two ways: for tick-borne infections, five *B. burgdorferi*-infected nymphal ticks (or non-infected control ticks) were placed on the dorsal thoracic midline of mice and allowed to attach and feed to repletion. To generate host-adapted *B. burgdorferi*, SCID-mice were infected s.c. via syringe inoculation with 10^4^ *B. burgdorferi* spirochetes grown to mid-log phase (day 5 of culture) in 0.1 ml of sterile medium. For infection with host-adapted spirochetes, 3 mm^2^ punch biopsies from infected SCID mice were obtained from the hairless, ethanol-cleaned ear pinnae. Biopsies were transplanted subcutaneously on the lateral side of the right tarsal joint of recipient naïve C57BL/6 mice. Ear transplants contained a mean of 1.8×10^4^ spirochetes, based upon quantitative DNA analysis [32]. Control mice were transplanted at the same location with similar tissue from uninfected SCID mice (sham infection).

### Ethics statement

This study was carried out in strict accordance with the recommendations in the Guide for the Care and Use of Laboratory Animals of the National Institutes of Health. All protocols involving animals were approved by the Animal Use and Care Committee at UC Davis (Permit Number: #15330).

### Borrelia burgdorferi

A clonal strain of *B. burgdorferi* sensu stricto (cN40) was grown in modified Barbour-Stoenner-Kelly (BSK II) medium [33] at 33°C and enumerated with a Petroff-Hauser bacterial counting chamber (Baxter Scientific, McGaw Park, IL). Heat-inactivation of *B. burgdorferi* was done at 56°C for one hour followed by sonication. Aseptically collected samples of lymph nodes, spleen, inoculation site, and urinary bladder were taken at necropsy and cultured for 7 and 14 days in BSK II medium to assess the presence of spirochetes under dark field microscopy.

### Ticks

Uninfected larval *Ixodes scapularis* ticks were obtained from field-collected adults in southern Connecticut (kindly provided by Durland Fish, Yale University). All larvae for the experiments described in this study were derived from a single cohort. A sample of the cohort was confirmed to be *B. burgdorferi flaB* negative by PCR. To generate infected nymphs, larvae were allowed to engorge on C3H mice that had been infected with *B. burgdorferi* for 2 weeks following syringe inoculation, as described previously [34]. Following feeding and molting, cohort analysis of the infected nymphal ticks revealed that 97% of the ticks were confirmed to be PCR positive for *B. burgdorferi flaB* as previously described [34].

### Histology and immunohistochemistry

Lymph nodes were fixed in neutral buffered formalin, embedded in paraffin and sectioned at 4 mm and stained with hematoxylin and eosin or by immunohistochemistry. Sections for immunohistochemistry were processed at room temperature and placed on positively charged slides, air-dried, de-paraffinazed and re-hydrated. Endogenous peroxidase activity was eliminated by incubation in 3% H_2_O_2_ in methanol for 20 minutes. Non-specific binding was reduced with biotin blocking solution (Vector) for 15 minutes and Power Block (InnoGenex) for 15 minutes. Immunohistochemical labeling of *B. burgdorferi* was performed by treating sections with 0.5 mg/ml protease type VIII (Sigma Aldrich) for 10 minutes, followed by 30 minutes incubation with 1∶1000 dilution of a polyclonal immune serum from *B. burgdorferi*-infected rabbits (infected for two months following inoculation with 10^4^ spirochetes). Antigen detection utilized a three-step streptavidin-horseradish peroxidase technique with the substrate DAB (Vector). For other antigens, antigen retrieval was enhanced by microwaving tissue sections for 6 minutes in citrate buffer at pH 6.0. Sections were then incubated with antibodies to B220 (CD45R, RA3-6B2), CD138 (281-2, BD Biosciences), or Ki-67 (NeoMarkers), followed by incubation with biotinylated secondary antibodies (Vector), streptavidin conjugated Alexa 488 and Alexa 594 (Molecular Probes) or streptavidin-horseradish peroxidase followed by DAB (Vector), and mounting with Prolong Antifade (Molecular Probes).

### Flow Cytometry

Live cell counts of single cell suspensions of lymph nodes were obtained using a hemocytometer and trypan blue exclusion of non-viable cells. Staining was performed using aliquots of 6.25×10^5^ cells in “staining medium” (buffered saline solution: 0.168 M NaCl, 0.168 M KCl, 0.112 M CaCl_2_, 0.168 M MsSO_4_, 0.168 M KH_2_PO_4_, 0.112 M K_2_HPO_4_, 0.336 M HEPES, 0.336 M NaOH, containing 3.5% heat-inactivated, filtered newborn calf serum and 1 mM EDTA) for 20 min on ice. The following antibody-conjugates were used at previously determined optimal concentrations: CD19-Cy5PE, CD3-APC Efluor780 (both e-biosciences), CD4-FITC, and CD8a-Cy5.5PE (both in-house generated) after blocking Fc receptor with anti-CD16/32 (2.4G2). Dead cells were discriminated with a live/dead violet staining kit (Invitrogen). Data acquisition was performed on a 13-color FACSAria instrument (BD Biosciences) [35]. Data were analyzed using FlowJo software (kind gift from Tree Star Inc.).

### Elispot

To probe for *B. burgdorferi*-specific antibody-producing cells by ELISPOT, 96-well plates (#MAHAS4510, Mixed Cellulose Ester Membrane; Millipore) were coated with 2.5 µg/mL of four recombinant non-lipidated *B. burgdorferi* N40 proteins: decorin binding protein A (DbpA), outer surface protein C (OspC), arthritis-related protein (Arp), and borrelia membrane protein A (BmpA) in PBS overnight. After blocking with PBS/4% BSA, lymph node cell suspensions were 2-fold serially diluted in medium (RPMI 1640, 292 µg/mL L- glutamine, 100 µg/mL of penicillin and streptomycin, 10% heat inactivated FCS and 0.03 M 2-ME) and cultured overnight at 37°C with 5% CO_2_. Cells were lysed with water and binding was revealed by incubation with biotin conjugated anti-IgM (Southern Biotech) or anti-IgH+L (Southern Biotech) for 2 hours in 2% BSA in PBS. This was followed by SA-HRP incubation for 1 hour (Vector Laboratories) in PBS/2% BSA and by 3-amino-9-ethylcarbazole (Sigma-Aldrich). Plates were washed and dried and mean spots were counted in all wells with visible spots and calculated as mean spot numbers per input cell number.

### Expression and purification of recombinant proteins

Genes encoding non-lipidated *B. burgdorferi* N40 proteins, previously identified by genomic expression library analysis to react with serum from *B. burgdorferi*-infected mice, as described [36], were amplified by PCR from *B.burgdorferi* N40 DNA using oligonucleotide primers based on their DNA sequences (Supplemental Table S1). Template DNA from the original reactive clone was denatured at 94°C for 1 min, annealed at 55°C for 1 min, and extended at 72°C for 1 min. This process was repeated for 30 cycles. The amplified genes were cloned in frame with the glutathione S-transferase (GT) gene into pMX, derived from a pGEX-2T vector (Pharmacia, Piscataway, N.J.) with a modified polylinker. The PCR-amplified DNA sequences were confirmed by sequence comparison with the original inserts.

*E. coli* DH5α cells transformed with the recombinant pMX vectors were grown to an optical density of 0.5 at 600 nm and the recombinant GT fusion proteins were induced with 1 mM IPTG for 2 h. Bacteria were centrifuged at 3,310 g for 20 min, pellets were washed with PBS and bacteria lysed with PBS/1% Triton X-100. The mixtures were sonicated and centrifuged at 35,000 g. Supernatants containing recombinant proteins were loaded onto glutathione-Sepharose 4B columns (Pharmacia), 25 U of thrombin was added to remove the GT partner, and purified proteins were eluted after 2 h.

### *B. burgdorferi* lysate preparation

*B. burgdorferi* was grown to log-phase (8–10 days), pelletted by centrifugation, resuspended in cold PBS plus MgCl_2_ and centrifuged repeatedly for 5 min at 4°C 17,500 g. Samples were stored in aliquots at −20°C. Protein concentration was determined using Bradford assay (Bio-Rad).

### Hybridoma generation

B cell hybridomas were created from enlarged lymph nodes collected at various times after infection with host-adapted *B. burgdorferi*. Three independent fusions were performed using standard protocols. Briefly, single cell suspensions from mechanically disrupted lymph nodes were fused with P3-X63Ag8.653 mouse myeloma cells (ATCC CRL-1580) using PEG 1450 (ATCC). Hybridomas were selected by incubating cells in HAT medium. Supernatants of all wells with visible cell growths were screened by ELISA for the presence of mouse Ig as previously described [37]. Hybridoma lines were established from all Ig-producers and tested further for reactivity against *B. burgdorferi*- specific recombinant antigens and whole *B. burgdorferi* lysate. Some hybridomas were then subcloned. The Ig heavy and light chain isotype profiles of the lines and clones were determined using the Mouse Immunoglobulin Cytometric Bead Array Kit, (BD Biosciences, Cat Number 550026).

### Statistical analysis

Statistical analysis was performed using the two-way ANOVA or Student's t-test with help of Prism 5 software (GraphPad Software). A *p*-value of <0.05 was considered statistically significant.

## Results

### Mice infested with *B. burgdorferi*-infected ticks develop regional and distant lymphadenopathy

Since lymphadenopathy has not been documented in laboratory mice following *B. burgdorferi* infection, we first sought to determine if and when lymphadenopathy developed in laboratory mice infected experimentally with *B. burgdorferi* via the natural route, i.e. via tick-bite. For that, *B. burgdorferi-*genetically susceptible C3H/He mice [38] , were each infested with either 5 infected nymphal ticks or with 5 uninfected nymphal ticks (sham-infected). All ticks were placed on the dorsal cervico-thoracic midline. However, the ticks subsequently migrated and attached to different regions of the body, particularly the head and neck region. The most common tick attachment sites were the ear pinnae and face.

**Figure 1:**
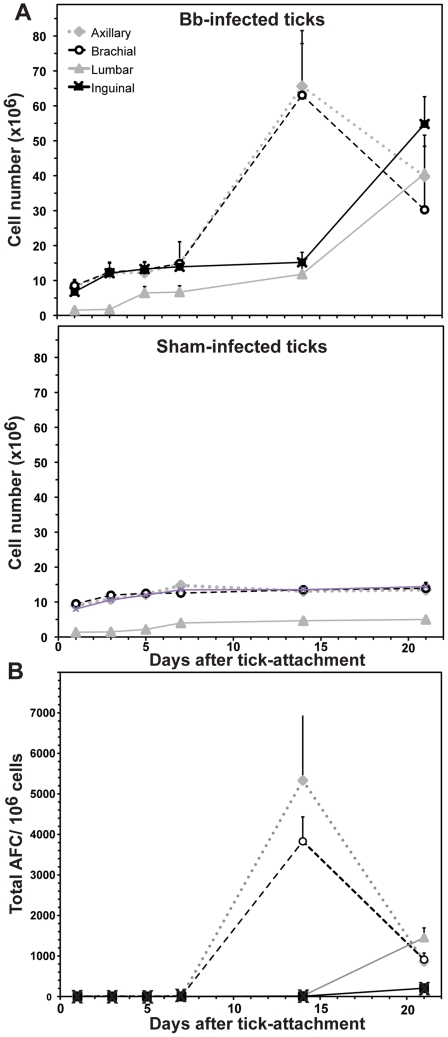
Tick-borne infection with *B. burgdorferi* causes systemic lymphadenopathy in mice. C3H/HeN mice (n = 30) were each exposed to five *B. burgdorferi*-infected nymphal *Ixodes scapularis* ticks (or non-infected ticks). At indicated times after tick-attachment, groups of five mice were necropsied and (**A**) cellularity of four indicated lymph nodes and (**B**) total antibody-forming cells (AFC) of these lymph nodes were assessed by hemocytometer count and ELISPOT analysis, respectively. Shown are mean values ± SD per timepoint for each lymph node type. ELISPOT analysis of lymph nodes from sham-infected mice showed no significant induction of antibody production (data not shown).

Axillary, brachial, lumbar and inguinal lymph nodes, among others, were collected at various times after infection and examined for visible signs of enlargement (not shown) and to determine cell number counts. Lymph node enlargement was noticed for all lymph nodes from mice exposed to *B. burgdorferi* infected ticks but not uninfected ticks (Figure 1A and data not shown). By day 14 following infestation with infected ticks, the lymph nodes closest to the tick-attachment site (axillary and brachial) were visibly enlarged and contained significantly increased numbers of cells in comparison to the same lymph nodes collected from the sham-exposed mice. Lymph nodes more distant from the attachment site (inguinal and lumbar) showed a slightly delayed increase in cellularity (Figure 1A). Thus, infection of laboratory mice with tick-borne *B. burgdorferi* faithfully recapitulates the lymphadenopathy observed in naturally infected humans and dogs, and suggests a relationship between time of lymph node enlargement and proximity to the site of infection.

### Rapid lymphadenopathy in regional lymph nodes of mice infected with host-adapted spirochetes

To directly assess the spatio-temporal relationship between the kinetics of the lymph node enlargement and the site of *B. burgdorferi* infection, a different infection modality was needed. Ticks change their attachment location in ways that varied significantly between mice, precluding targeted analysis of specific lymph nodes. Direct inoculation of mice with culture-grown *B. burgdorferi*, on the other hand, introduces untoward experimental variables due to the significant antigenic changes that *B. burgdorferi* undergoes as it adapts to the vertebrate host. One example is the antibody-response to the major outer surface protein A (OspA), which is strongly expressed in vitro and in ticks [39], but virtually absent in mice infected with *B. burgdorferi* via tick-infestation or following transplantation of tissue from infected mice containing host-adapted spirochetes [27]. Thus, infection via injection of culture-grown bacteria may favor distinct immune responses that differ from those seen after tick-infection. We therefore transplanted punch biopsies of ear pinnae from infected SCID mice, containing host-adapted spirochetes under the skin of the right tibiotarsus area of congenic, naïve C57BL/6 mice. The right inguinal lymph nodes were evaluated as the regional lymph nodes.

**Figure 2:**
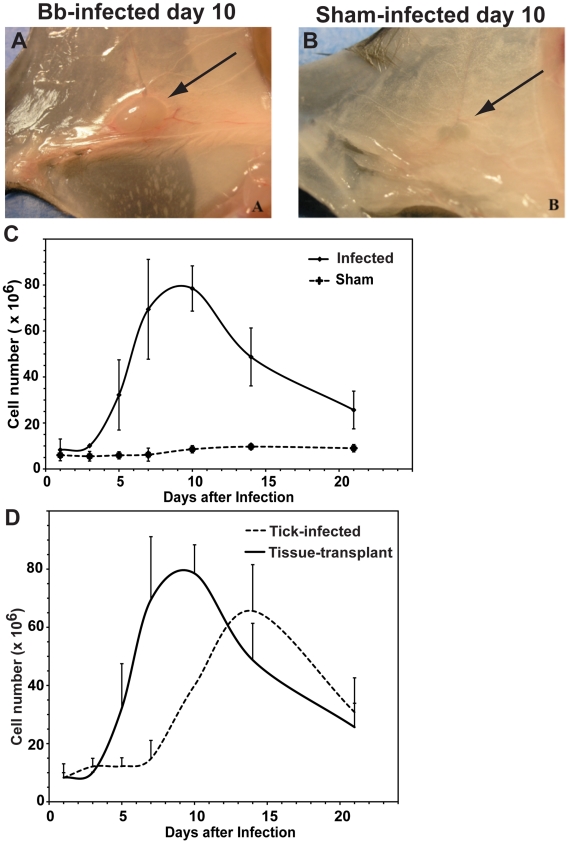
Infection with host-adapted *B. burgdorferi* induces lymphadenopathy near the site of infection. Lymphadenopathy of the right inguinal lymph node of C57BL/6 mice 10 days after infection via subcutaneous transplantation of small pieces of ear from *B. burgdorferi*-infected (**A**) or non-infected (**B**) congenic SCID mice into their right dorsal tarsal region. (**C**) Shown are mean cell numbers ± SD of right inguinal lymph nodes from groups of four mice per timepoint. (**D**) Comparison of lymph node celluarity (mean ± SD) obtained from draining lymph nodes in (C) and from the axillary lymph nodes of tick-infected mice as shown in Figure 1.

Infection of C57BL/6 mice with host-adapted *B. burgdorferi* resulted in a rapid enlargement of their regional inguinal lymph nodes (Figures 2A, 2B). These increases closely resembled the lymphadenopathy observed at the site of tick-attachment following tick-borne infection, albeit with somewhat faster kinetics (Figure 2C), possibly due to the increased time between tick-attachment and actual infection, and/or the time it takes for *B. burgdorferi* to adapt to the host-environment prior to dissemination [34]. Similar to tick-borne infection, infection with host-adapted spirochetes caused a generalized lymphadenopathy, with lymph nodes more distant from the infection-site increasing slower in cell numbers compared to those closest to the site of infection (Table 1). Thus, this infection model faithfully recapitulated tick-borne *B. burgdorferi*-induced lymphadenopathy with the advantage that we can consistently identify the lymph nodes draining the site of infection. The spleen was not increased in size or cellularity following either tick-borne infection (not shown) or following infection with host-adapted spirochetes (Table 1).

**Table 1:** *B. burgdorferi* presence correlates with lymphadenopathy in mice infected with host-adapted spirochetes.

	*Right Inguinal*		*Lumbar*		*Right Axillary*		*Left Inguinal*		*Left Axillary*		*Spleen*	
	Bb*	Cells†	Bb	Cells	Bb	Cells	Bb	Cells	Bb	Cells	Bb	Cells
Day 0	−	5.4	−	3.2	−	3.5	−	7.6	−	5.2	−	102
Day 1	+	4.6	−	3.8	−	3.2	−	4.1	−	3.7	−	112
Day 2	+	6.1	+	4.5	−	3.6	−	5.4	−	4.1	−	105
Day 3	+	10.2	+	4.8	−	4.6	−	5.3	−	3.1	−	133
Day 4	+	8.2	+	4.6	+	5.1	−	4.9	−	3.5	−	98
Day 5	+	6.6	+	3.7	+	6.4	+	5	−	3.2	−	123
Day 7	+	**57**	+	5.1	+	3.2	+	2.8	+	1.7	−	106
Day 10	+	**79**	+	**56.2**	+	**46**	+	**19**	+	**25**	+	95
Day 14	+	20.2	+	**41.3**	+	**62**	+	**44**	+	**54**	−	132
Day 21	+	21	+	18.8	+	14.6	+	12	+	11.4	−	121
Day 28	+	16.8	+	9.2	+	12.9	+	14.4	+	10.6	−	117
Day 60	+	11.4	+	5.7	+	6.4	+	10.1	+	8.6	+	147
Day 90	+	15.4	+	6.4	+	7.4	+	11.9	+	6.2	+	138

*Cultures on lymph nodes assessed at 1 and 2 weeks. “+” indicates 4/4 positive and “–” indicates 0/4 negative culture result for *B. burgdorferi* (Bb).

**†:** Mean number of total cells (×10^6^) of live cells from C57BL/6 mice (n = 4) counted via a hemocytometer using trypan blue exclusion**.** Numbers in bold indicate peak of lymph node cellularity.

### Lymphadenopathy is caused by the presence of extracellular *B. burgdorferi* in the lymph nodes

Since proximity to the infection site was correlated with an increase in lymph node cellularity, we investigated next if and when *B. burgdorferi* could be cultured from the lymph nodes. Within 24 h following infection with host-adapted spirochetes, *B. burgdorferi* was cultured from the closest draining right inguinal lymph nodes, but not any other lymph nodes (Table 1). By 48 h, the right lumbar lymph nodes became culture-positive. The right axillary lymph nodes yielded positive culture results two days later and before any of the contralateral lymph nodes on the left side of the mouse. A few days after the lymph nodes became culture-positive (about 4–6 days), a marked increase in cellularity of the lymph nodes was consistently observed for all lymph nodes, but not to the degree as the most proximal regional lymph nodes (Table 1). Once culture-positive, the lymph nodes remained so for the 90-day study period. Culture results from the spleen did not reveal *B. burgdorferi* until day 10 and then also only intermittently thereafter (Table 1).

**Figure 3:**
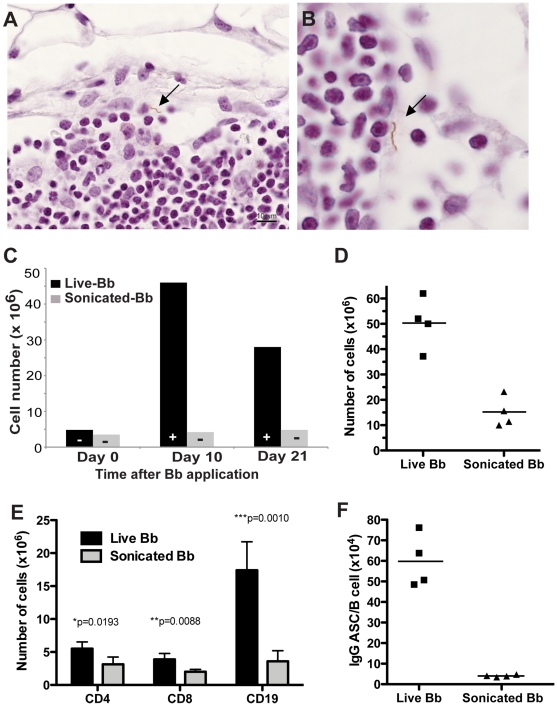
*B. burgdorferi* localizes to the subcapsular sinus of lymph nodes. (**A, B**) Immunohistochemistry with polyclonal *B. burgdorferi*-specific rabbit serum, demonstrates the presence of *extracellular B. burgdorferi* spirochete (arrow) in the subcapsular sinus of the right inguinal lymph node of C57BL/6 mice infected by tissue-transplant eight days prior to analysis. (**C**) Shown are mean cell numbers of right inguinal lymph nodes of C57BL/6 mice (n = 2 per timepoint) either infected via injection of 10^4^ culture-grown live (live) or heat-killed and sonicated *B. burgdorferi* in PBS. “+” identifies positive *B. burgdorferi* cultures of lymph nodes (n = 2 per timepoint) from the same group of mice, “−” shows lack of *B. burgdorferi* growth in culture. Results are from one of two independent experiments that gave similar results. (**D**–**F**) Comparison of mice infected as in (C) but injected with 10^6^ inactivated *B. burgdorferi* for 8 days prior to analysis (**D**) Total numbers of cells recovered from lymph nodes. Each symbol represents results from one animal; horizontal bars indicate the mean for the group. (**E**) Data shown are total numbers CD4, CD8 T cells and CD19 B cells calculated from total cell counts and frequencies of cell subsets as determined by flow cytometry. (**F**) Shown are frequencies of IgG antibody-secreting cells in lymph nodes of mice given live or inactivated *B. burgdorferi*. Note data are normalized to B cell numbers. Each symbol represents the results from one mouse; the horizontal line indicates the mean for the group.

These data suggested that the lymphadenopathy observed during Lyme borrreliosis is caused by a massive increase in lymph node cellularity triggered by the accumulation of live *B. burdorferi* spirochetes into the lymph nodes. Alternatively, it was possible that the culture results were a mere reflection of the presence of *B. burgdorferi* in the lymph node capsule, given that the spirochete travels along connective tissues. In that case, the increase in lymph node cellularity would be an indirect consequence of the infection-induced inflammation rather than the presence of the spirochetes in the lymph nodes. To distinguish between these possibilities, immunohistochemistry was utilized to determine the precise tissue-location of the spirochetes in lymph nodes of mice infected for 8 days with host-adapted *B. burgdorferi*. The results demonstrated the consistent presence of *B. burgdorferi* spirochetes in the sub-capsular sinus and superficial cortex of infected lymph nodes (Figures 3A, 3B). Interestingly, the spirochetes were found in the lymph nodes extracellularly appeared intact with characteristic spiral morphology. Together with the results from the culture experiments (Table 1), this suggests a degree of persistence of *B. burgdorferi* in lymph nodes.

Because lymphadenopathy was correlated with the presence of viable spirochetes, we determined next if lymphadenopathy could also be induced with inactivated spirochetes. For that, mice were either infected directly by subcutaneous inoculation in the right tarsal region with 10^4^ viable cultured spirochetes or by the same number of spirochetes after inactivation by sonication. Inactivation was confirmed by culture of the sonicate. Right inguinal lymph nodes were collected at days 0, 10 and 21 days post infection and either cultured in BSK II medium or assessed for cellularity. At days 10 and 21 post infection, *B. burgdorferi* was cultured from the right inguinal lymph node of mice infected with live spirochetes but as expected, not from mice inoculated with inactivated spirochetes. Importantly, increases in lymph node cellularity were not observed in mice receiving inactivated *B. burgdorferi*, but were clearly induced in mice inoculated with viable *B. burgdorferi* (Figure 3C).

Since *B. burgdorferi* might replicate *in vivo* and thus the results might reflect application of differing amounts of bacteria or bacterial antigen between these two groups, the analysis was repeated by giving 100-fold higher amounts of inactivated bacteria (10^6^ organisms). While the increased amount of inactivated bacteria resulted in lymph node enlargement compared to control mice (Figures 3C, 3D), the enlargement was significantly less (p = 0.001) than that seen with viable Borrelia (Figure 3D). Thus, we conclude that lymphadenopathy during *B. burgdorferi* infection is caused by the accumulation of viable spirochetes in lymph nodes.

### Increase in lymph node cellularity is due to massive expansion of B cells

**Figure 4:**
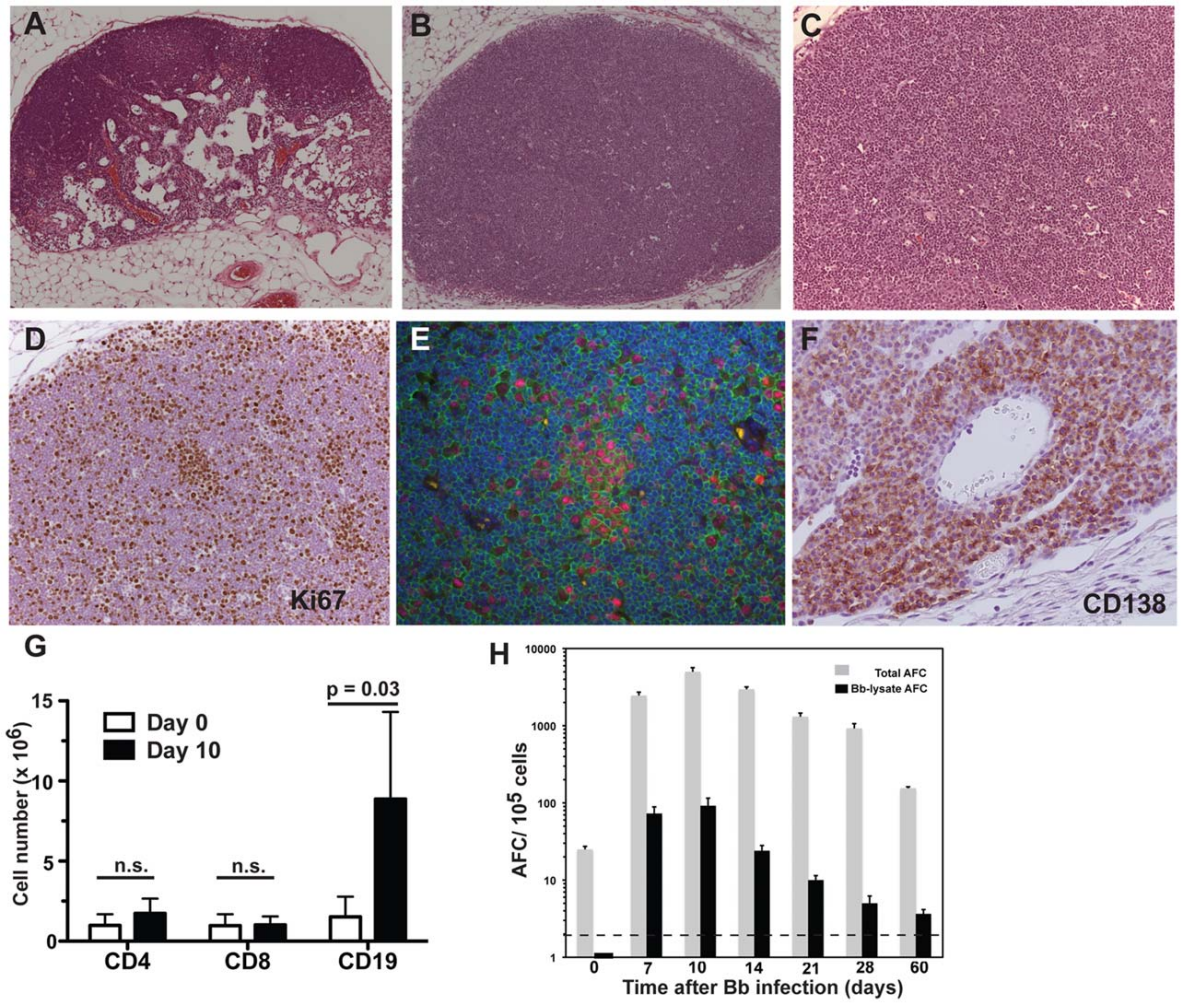
*B. burgdorferi*-induced lymphadenopathy is caused by a massive proliferation and differentiation of lymph node B cells. (**A–C**) H.E. staining of right inguinal lymph node from a C57BL/6 mouse (**A**) sham-infected or (**B**) infected by tissue-transplantation with host-adapted spirochetes 10 days prior. (**C**) Close-up image of (B). Note the complete lack of follicular and extrafollicular structure. (**D**) Immunohistochemistry on lymph nodes as in (B, C) reveals the presence of large numbers of Ki67+ proliferating cells in the lymph node cortex. (**E**) Three-color immunofluorescence identifies the Ki67+ (red) cells as B220+ (green) B cells. Blue color indicates nuclei as stained with DAPI. (**F**) Large numbers CD138+ plasma cells accumulate in the medullary cords of lymph nodes of infected mice as (in B/C) (**G**) Shown are mean cell numbers ± SD from 4 C57BL/6 mice per time point of CD4, CD8 T cells and CD19+ B cells in inguinal lymph nodes before (white) and at day 10 (black) of infection. Data were calculated from flow cytometric evaluation of live cell frequencies and total live cell counts by hemocytometer with trypan blue exclusion. Results are from one of >3 experiments performed that gave similar results. Statistical analysis was conducted by comparing results from each cell population using Student's *t* test; n.s. not significant. (**H**) Shown are mean numbers of antibody-secreting cells (AFC) ± SD for total (grey bars) and Borrelia-lysate (black bars)-specific antibodies in inguinal lymph nodes from 4 C57BL/6 mice per timepoint at the indicated times after infection with host-adapted *B. burgdorferi*. Horizontal bar indicates threshold levels of detection.

Next, the cause of the increase in cellularity of the lymph nodes was investigated. Immunohistochemistry on day 10 after infection demonstrated stark differences in lymph node organization compared to lymph nodes from uninfected mice (Figures 4A, 4B). Morphologically, the cortex of infected regional lymph nodes consisted of tightly packed extrafollicular lymphocytes and very few scattered, poorly demarcated germinal centers without a distinct mantel zone. Indeed follicular structures appear largely absent in these lymph nodes (Figures 4B, 4C). The majority of the lymphocytes in the cortex were positively labeled by B220 and thus identified as B cells (data not shown). The largest extrafollicular B cells were frequently arranged in distinct clusters interpreted as antibody forming foci. These large B cells were characterized by an open euchromatic nucleus with marginated chromatin and a large prominent nucleolus and moderate amounts of cytoplasm, characteristics of plasmablasts.

To distinguish between increased trapping of migrating cells versus expansion of cells within the regional lymph nodes, cells were labeled for Ki-67 antigen to identify proliferating cells. The staining demonstrated large numbers of B cells in the cortex that were actively dividing (Figure 4D). Cells in the paracortex that were negative for B220 rarely expressed Ki-67, suggesting that cell division was restricted to the B cell population only (data not shown). Dual fluorescent labeling of Ki-67 and B220 identified the dividing cells as B cells (Figure 4E). The exclusive and extensive expansion of B cells was further confirmed by flow cytometry. Whereas the lymph nodes showed no significant increases in either CD4 or CD8 T cells compared to lymph nodes from non-infected mice, CD19+ B cells had expanded dramatically by day 10 of infection with live *B. burgdorferi* (Figure 4G). The massive increases in B cells were absent in mice injected with inactivated *B. burgdorferi* (Figure 3E).

In addition, the medullary cords of the lymph nodes from the infected mice showed the presence of large numbers of plasma cells, identified by staining with CD138 (Figure 4F), suggesting a strong induction of antibody secretion in the affected lymph nodes. Indeed, ELISPOT analysis on regional lymph nodes infected for up to 60 days with *B. burgdorferi* showed the presence of large numbers of antibody-forming cells (AFC), with peak responses noted around day 10 of infection (Figure 4H). Depending on the day of study between 1–3% of the AFC secreted antibodies were bound to the Borrelia lysate (Figure 4H). The strong antibody secretion within the lymph nodes following infection with host-adapted spirochetes was in magnitude and kinetics very similar to the induction seen following tick-borne infection with *B. burgdorferi* (Figure 1B), but was much larger than seen after injection of inactivated bacteria (Figure 3F). In summary, expansion of the lymph node cortex by reactive B cells and extrafollicular antibody forming foci constitutes the morphological basis of lymphadenopathy in Lyme borreliosis.

### B cell expansion and differentiation following infection with *B. burgdorferi* is at least in part antigen-specific

The finding of active migration of *B. burgdorferi* into lymph nodes, i.e. an organ responsible for immune response induction, appeared counter-intuitive for an organism that aims to establish persistent infection. Therefore, we aimed to determine next whether *B. burgdorferi* might cause immune subversion in these lymph nodes. In particular we asked whether it was inducing massive non-specific B cell expansion and differentiation to antibody-secreting cells at the expense of an effective Borrelia-specific antibody response. Addressing this question is complicated by the fact that protein expression of culture-grown spirochetes does not fully resemble Borrelia in the host, i.e. the usefulness of protein lysates from culture-grown bacteria is limited as a source of antigen for ELISPOT analysis.

**Figure 5:**
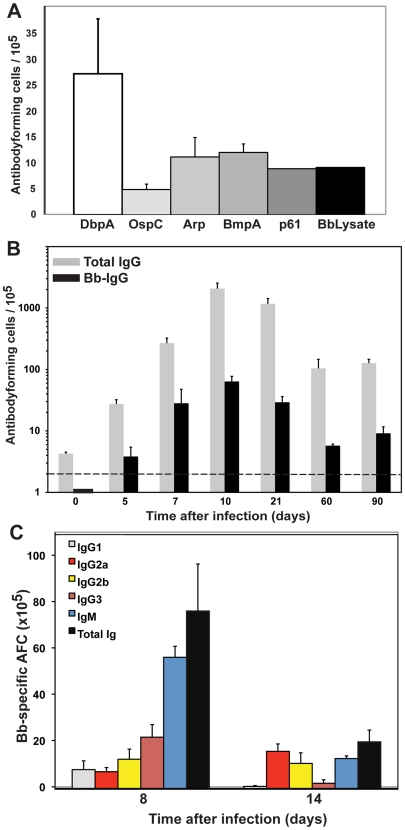
Strong induction of *B. burgdorferi*-specific antibodies following infection. (**A**) Indicated recombinant *B. burgdorferi* antigens and lysate were tested as coating reagents for ELISPOT to enumerate *B. burgdoferi*-specific antibody-secreting cells (AFC) in the inguinal lymph nodes of C57BL/6 mice infected for 14 days with host-adapted *B. burgdorferi*. Shown are mean numbers of antibody-secreting cells (AFC) ± SD from 5 mice. (**B**) A pool of recombinant proteins (DbpA, OspC, Arp and BmpA) was used to determine the frequencies of *B. burgdorferi*-specific antibody-secreting cells (black bars) by ELISPOT in C57BL/6 mice at indicated times after infection. Grey bars indicate the total number of IgG-AFC per lymph node. For each timepoint two infected mice and two sham-infected control mice were analyzed. Shown are the mean numbers *B. burgdorferi*-specific antibody-secreting cells ± SD assessed for the individual mice from which cell spots seen in sham-infected mice were subtracted. SD was calculated from titration curves of 3 replicates for each of the two mice analyzed. (**C**) Shown are the mean frequencies ± SD of *B. burgdorferi*-specific antibody-secreting cells expressing the indicated Ig-isoytypes or total Ig (black) on days 8 and 14 after infection. Data are from 4 mice per group and timepoint.

Initial studies were therefore conducted to identify a number of Borrelia antigens that are expressed in the host and induce robust antibody responses. A screen of available recombinant Borrelia-expressed antigens by ELISPOT analysis with lymph node cells from day 14 Borrelia-infected mice showed that lymph nodes had measurable reactivity against all of the recombinant antigens tested. Interestingly, DbpA had the highest level of reactivity, while the Borrelia lysate, included as a “positive” control, identified a much smaller fraction of Borrelia-reactive AFC (Figure 5A). Further analysis showed that it was possible to pool various Borrelia antigens for ELISPOT analysis without losing sensitivity of reactivity against each antigen (data not shown). A pool of four recombinant antigens, consisting of DbpA, OspC, Arp, BmpA was used as a means of measuring the Borrelia-specific antibody response. While it clearly underestimates the number of total Borrelia-specific responses, testing with the pool of recombinant proteins that are expressed during infection was found to be more sensitive than testing with Borrelia lysate.

A time course analysis of C57BL/6 mice infected for up to 90 days with host-adapted *B. burgdorferi* showed the robust induction of antibody-secretion within the regional lymph nodes (Figure 5B). The kinetics of the Borrelia-specific response was identical to that of the total antibody responses measured at the site (compare Figure 4H with Figure 5B). Depending on the day of analysis between 4–13% of AFC were shown to be specific for one of the four recombinant proteins included as antigens in the analysis. The isotype profile of the specific response showed a broad representation of all measured isotypes. More than half of the antibody-secreting cells appeared to generate IgM antibodies and IgG antibody isotypes classically associated with T-independent responses (IgG2b and IgG3, Figure 5C). Overall, the isotype profile of the Borrelia-specific response suggested that a considerable proportion of the B cell response might be T-independent, consistent with previous observations [29], [40].

### Borrelia-specific B cell responses are strongly induced in regional lymph nodes following infection

**Figure 6:**
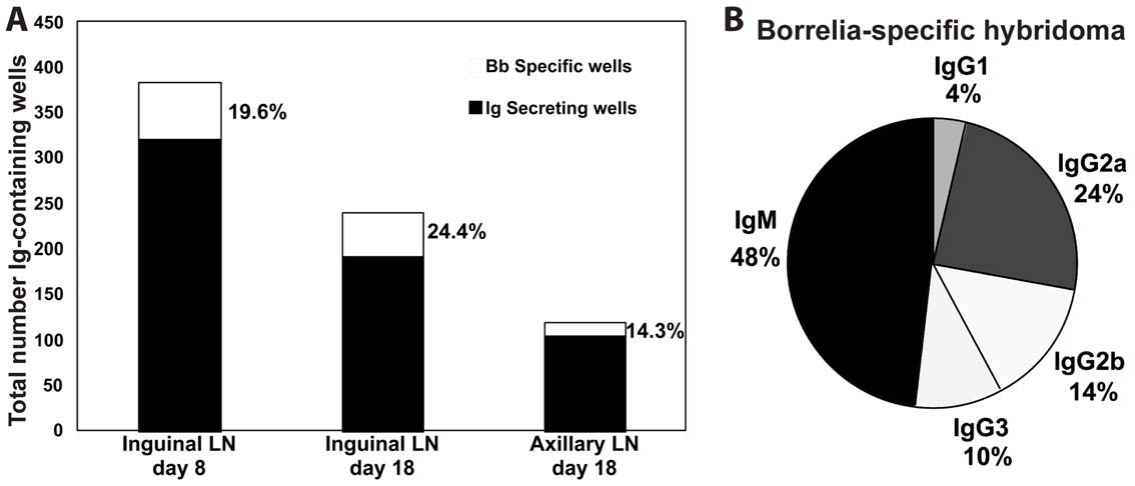
Lymph nodes of *B. burgdorferi*-infected mice contain large numbers borrelia-specific B cells. Shown are the results of three independent hybridoma fusion experiments. (A) Shown are stacked bars with the total number of antibody-secreting cells (black) and the number of lines generating *B. burgdorferi*-specific antibody as assessed by ELISA with four recombinant *B. Burgdorferi* antigens. Cells for the fusion were from indicated lymph nodes and times after infection with host-adapted spirochetes. (B) Indicated are the Ig-isotype distribution among all *B. burgdorferi*-specific hybridoma lines generated.

The ELISPOT results suggested that a significant fraction of the induced B cell response was specific and directed against *B. burgdorferi*. However, given that we probed with only some of the many other Borrelia proteins that are potentially expressed selectively *in vivo*, assessment of the relative contribution of the specific over the non-specific response was difficult. Therefore, another series of experiments was conducted in which hybridomas were generated from the regional lymph nodes to assess the fraction of hybridomas directed against Borrelia-specific antigens with an expanded list of recombinant Borrelia proteins. Three successful fusions were conducted, including one on lymph nodes at day 8 of infection, and two on day 18. The overall results from these three fusions were similar (Figure 6A). Initial screening of roughly 1000 wells per fusion identified between 150–350 wells that showed antibody-secretion. Further screening of the antibody-secreting lines indicated that between 14–24% of the hybridoma lines generated antibodies that could be identified to react against an expanded list of recombinant Borrelia antigens (DbpA, OspC, Arp, BmpA, P23, P29, P32, P61 (defined in Supplemental Table S1) [41] and/or Borrelia lysate from cultured spirochetes.

From these fusions, 132 hybridoma lines were cultivated. Of these, 45 were specific for *B. burgdorferi* antigens. The isotype profile of the 45 hybridoma lines matched very closely that observed by ELISPOT on Borrelia-infected lymph nodes, indicating that the hybridoma lines were recapitulating the responses *in vivo* (Figure 6B). Furthermore, the largest fraction of the 45 Borrelia-specific hybridomas (11/45) recognized DbpA (data not shown). This is consistent with the initial specificity screen by ELISPOT (Figure 5A). Given that the recombinant antigen pool was likely to underestimate the frequencies of antigen-specific B cells/hybridomas, we conclude that a sizable fraction of the massive B cell response induced during Lyme borreliosis is specific against *B. burgdorferi*.

### Lymphadenopathy and B cell induction are independent of MyD88

**Figure 7:**
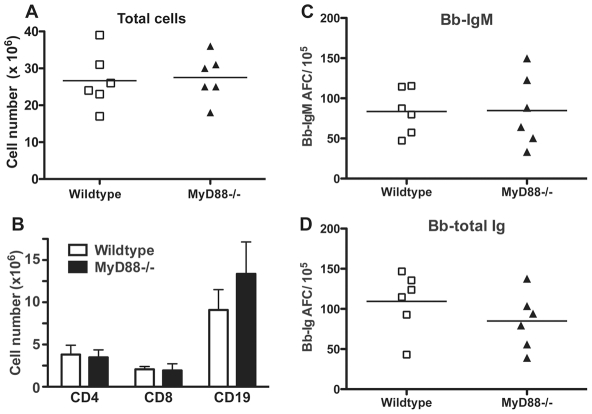
Lymphadenopathy and lymph node B cell activation are independent of MyD88-signaling. Control C57BL/6 (wildtype) and congenic MyD88−/− mice (n = 6 per group) were infected with host-adapted *B. burgdorferi* for 10 days. Lymph nodes were harvested and compared for (**A**) total cellularity and (**B**) numbers of CD4, CD8 and CD19 as assessed by flow cytometry. ELISPOT analysis was conducted to determine numbers of (**C**) borrelia-specific IgM or (**D**) all Ig-isotype secreting borrelia-specific cells. For scatter plots, each symbol represents the result from an individual animal. Lines indicate mean of the group. Bar chart shows the mean values ± SD. Results are pooled from two independent experiments. Statistical analysis for (A) (C) (D) was conducted by Student's *t* test and (B) by two-way ANOVA. None of the data showed significant differences between control and MyD88−/− mice.

Non-specific mitogenic stimulation of B cells with Borrelia lipoproteins *in vitro* has been reported previously [9]–[16]. OspA, a surface lipoprotein that is strongly expressed by Borrelia in culture, but down-regulated upon infection of a mammalian host, was shown to be responsible for at least some of the mitogenic activity [11], [14]. While host-adapted spirochetes are not expected to express significant amounts of OspA, other proteins or lipids may provide mitogenic signals to B cells *in vivo*. Therefore, we determined the role of the adaptor protein MyD88, important in TLR and IL-1-mediated innate signaling, in regulation of initial B cell activation and/or the lymph node enlargement. A previous study found impaired pathogen-clearance and alterations in the antibody-isotype profile of serum antibodies in mice lacking MyD88 [42]. MyD88−/− mice and congenic control mice were infected with host-adapted spirochetes for ten days. The analysis revealed no role for MyD88 in the quality or magnitude of the lymphadenopathy. Regional lymph nodes from MyD88−/− mice had similar cell numbers on day 10 of infection (Figure 7A), with similar predominance of CD19+ B cells compared to control mice (Figure 7B). Furthermore, there was no difference in the number of Borrelia-specific IgM or total Ig secreting cells in the lymph nodes (Figures 7C, 7D). Thus, MyD88-dependent innate signaling is not driving the induction of lymphadenopathy, nor the massive activation of B cell responses associated with Lyme borreliosis. Together with the strong antigen-specific B cell responses measured by hybridoma generation, the results suggest that Borrelia-infection induces a specific, albeit largely extrafollicular B cell response as a result of the accumulation of live *B. burgdorferi* in lymph nodes.

## Discussion

This study provides new insights into the pathogenesis of lymphadenopathy during the early stages of human Lyme borreliosis. The results demonstrate for the first time the extracellular accumulation of *B. burgdorferi* in the cortical regions of lymph nodes and implicate the direct association of migrating *B. burgdorferi* spirochetes with a marked and specific but unusual B cell response in the lymph nodes, but not the spleens, of mice infected with tick-borne or host-adapted spirochetes. The strong accumulation of proliferating B cells in the cortical areas of the lymph nodes, in the absence of a simultaneous accumulation of CD4 T cells (Figures 3, 4), the lack of strongly demarcated lymph node follicles and germinal centers in the lymph nodes (Figures 4B, 4C), and the strongly IgM and IgG3/2b-driven specific antibody response (Figures 5, 6) indicate that this pathogen drives the borrelia-specific B cell response towards T cell-independence. From these results we hypothesize that these effects of *B. burgdorferi* on the Borrelia-specific B cell response constitute a novel immune-evasion strategy.

The active migration of *B. burgdorferi* into sites of immune induction appears counter-intuitive for an organism that aims to establish persistence. Their presence in the lymph nodes and the strong responses their presence evokes thus indicate the intricate balance this pathogen achieves between immune induction and immune evasion. The nature of the observed B cell response is clearly distinct from that observed following acute infections with other non-persistent pathogens, or following immunizations with various protein antigens that induce mainly T cell-dependent extrafollicular and germinal center responses [43], [44]. In particular we note a lack of clearly demarcated follicles, with it an apparent lack of germinal centers and the accumulation of proliferating B cells in the follicles. The presence of T-independent B cell responses to *B. burgdorferi* had previously been indicated by measurements of strong antibody-responses in the serum of T cell-deficient mice [29]. While we cannot fully exclude the possibility that it is the nature of the expressed Borrelia-antigens that drive the B cell response towards its extrafolicular nature (Figure 4C) and apparent T-independence, we believe that this cannot fully explain our observations. A major component of the B cell response induced to infection with host-adapted spirochetes was directed against decorin-binding protein (DbpA) (Figures 5, 6). When administered in adjuvant, a strong germinal center-response was observed in the draining lymph nodes and DbpA-specific antibody responses were strongly induced against this protein, suggesting that this major *B. burgdorferi* immunogen is capable of inducing T-dependent responses in the right context (unpubl. observations). Furthermore, immunization with DbpA does induce protective antibody responses [36].

Also, the strongly B cell-driven lymphadenopathy seen following *B. burgdorferi* infection was not observed following immunization of mice with culture-grown heat-killed and sonicated spirochetes (Figures 3C, 3E), although such immunization increased both lymph node size (Figure 3D) and induced moderate frequencies of *B. burgdorferi*-specific antibody-secreting cells (Figure 3F). Thus, either live infection and/or the presence of live extracellular bacteria and bacterial proteins in the cortex areas of the lymph nodes appear to trigger this unique B cell response to spirochetes, or alternatively, the response is triggered by an antigen(s) not present on the culture-grown bacteria used for immunization. Since neither the lymphadenopathy nor the B cell response were significantly different following infection of MyD88−/− mice compared to controls (Figure 7), TLR-mediated inflammatory responses can be excluded as potential triggers of this response, in contrast to apparently similar TLR-4-mediated alterations following *S. typhimurium* infection [17].

From this, it is tempting to speculate that it is the expression of specific immune-subversion antigens by *B. burgdorferi* in the mammalian host that induce overshooting and potentially aberrant T cell independent B cell responses that are neither of sufficient high-affinity nor induce memory responses able to combat primary and repeat infections. The analysis of candidate antigens must await the development of techniques that allow us to comprehensively compare protein expression by culture-grown and tissue-adapted spirochetes within the context of specific tissue sites, such as lymph nodes.

While the induced B cell response to *B. burgdorferi* is unable to clear the infection, it does provide immune protection from overt disease. This is indicated by studies in B cell- or CD40L-deficient mice that showed increased signs of tissue-inflammation and disease progression compared to controls [29], [40], [45]. Furthermore, passive transfer of immune serum from infected mice confers immune protection from infection when injected prior to pathogen challenge [26], [30]. Thus, understanding the mechanisms that induce and regulate the borrelia-specific B cell response is of importance. Assessing the specificity of the B cell response to *B. burgdorferi* is challenging, however, due to differences in the antigenic structure of *B. burgdorferi* cultured in artificial media versus those grown in the mammalian host [39], [46]–[50]. Thus, lysates or extracts from culture-grown spirochetes do not reflect antigens expressed in the mammalian host. Furthermore, *B. burgdorferi* differentially expresses antigens during the various stages of its life cycle in the flat tick, the feeding tick and the host [39] . We therefore utilized an infection protocol that mimics tick-borne infection and avoids induction of immune responses to Borrelia antigens not expressed *in vivo*, by infecting mice with mammalian host-adapted spirochetes via tissue transplant.

For detection of *B. burgdorferi*-specific antibodies by ELISA and ELISPOT, we used a cocktail of recombinant antigens, including OspC, DbpA, Arp and BmpA, each of which are expressed during infection of the mammalian host [36], [47], [49], [51]–[53]. Furthermore, each of these antigens have been shown to induce protective or disease-resolving immune responses in mice [41], [47], [54]–[57]. We did not include the VlsE protein in our studies, a surface-protein thought to subvert the immune response to *B. burgdorferi* through extensive genetic variation within the host. However, the N40 strain of *B. burgdorferi*, which we have used here, does not seem to express this protein, based on transcriptional analysis of the IR6 region of vlsE. Moreover, we found no evidence of seroconversion to the C6 antigen of vlsE from strain B31 (S. W. Barthold, unpublished). Recent sequence analysis of the N40 genome has confirmed that N40 vlsE and BBK01 are on different plasmids and that the vlsE locus is indeed significantly different compared to B31, the commonly used VlsE-expressing Borrelia-strain.

Using only a handful of such in vivo-expressed and immunodominant antigens, we demonstrated the induction of a strong *B. burgdorferi*-specific antibody response in the lymph nodes of infected mice (Figures 5, 6) in a manner that is independent of MyD88 (Figure 7). We furthermore showed that nearly a quarter of hybridomas generated from lymph nodes of acutely *B. burgdorferi*-infected mice are specific for this pathogen (Figure 6). Together with earlier studies that demonstrated the protective and disease-resolving capacity of immune sera from long-term infected mice [26], [30], [58], we can conclude that a strong and borrelia-specific B cell response is induced in these lymph nodes.

*B. burgdorferi* causes spirochetemia, but its primary means of dissemination is via migration through host connective tissues and extracellular matrix [59] . This is consistent with our finding of progressive involvement of the ipsilateral, but not the matching contralateral lymph nodes of the host (Table 1). Furthermore, the spleens of infected mice did not differ in size or in frequencies of *B. burgdorferi*-specific antibody-secreting cells compared to spleens from uninfected mice, and were only sporadically culture-positive for spirochetes (Table 1). In apparent contrast, a previous study in mice reported the involvement of marginal zone B cells in the response to *B. burgdorferi* infection [60]. This difference to our study might well be due to the difference in the route of infection, i.e. intra-cutaneously with culture-grown bacteria at the back of mouse versus infection with host-adapted spirochetes by tissue-transplantation in the tarsus region. It has been well documented that the course of infection and organ involvement varies with the site of inoculation in mice [61], [62]. Furthermore, it is notable that tick-borne infections also failed to induce a significant B cell response in the spleen (data not shown).

In conclusion, by accumulating in the extracellular cortical spaces of the lymph node, *B. burgdorferi* seems to both induce and subvert an important arm of the adaptive immune response. Rather than fully suppressing the activity of B cells, *B. burgdorferi* appears to shift the major B cell response towards the production of antibodies generated in extrafollicular foci. It thereby seems to support the production of antibodies that provide immune protection from disease, while subverting the induction of more strongly protective, possibly T-dependent B cell responses that could confer bacterial clearance.

## Supporting Information

Table S1 Primers for the generation of recombinant *Borrelia burgdorferi* N40 antigens used for detection of B cell responses.
(DOC)
