# Supplementary material for: Lymphoadenopathy during Lyme Borreliosis Is Caused by Spirochete Migration-Induced Specific B Cell Activation
Source: PLoS Pathog. 2011 May 26;7(5):e1002066. doi: 10.1371/journal.ppat.1002066 (PMC3102705; doi:10.1371/journal.ppat.1002066)
Supplement: Table S1 — Primers for the generation of recombinant Borrelia burgdorferi N40 antigens used for detection of B cell responses. (DOC) [file ppat.1002066.s001.doc]

**Supplemental Table 1: Primers for the generation of recombinant *Borrelia burgdorferi* N40 antigens used** for detection of B cell responses

| **N40**  **Protein** | **B31 homol.*** | **F primer** | **R primer** |
| --- | --- | --- | --- |
| Arp | BBF01 | AAATTTGATAGTCTTAATTTATCTACAAAAAGCGTAGAT | TTAACTTAAACCCTTTACACTTTCTTC |
| DbpA | BBA24 | GGATTAAAAGGAGAAACAAAAATCATATTA | GTTATTTTTGCATTTTTCATCAGTAAAATT |
| P23 | BBQ38 | ATGAAGTTTTCGGTTACTAATGCTAATGATGCA | TCAGCAGCCTTTATCATCCCTTAA |
| P61 | BB0329 | CTTGGGAGCAGAGCCAAGCAGTCTTGAC | GCTTTCTGCGATATTTGGTACCCACC |
| P29 | BB0811 | CAAAATGTTAATTCTCCAACTCTTCC | TGGGAAATGATGGTCAAAAATTAAGC |
| P27 | BBK47 | Gaattcatgtcagatgatcctggtgca | Ctcgagttaaagcttgaatatattttctatttcttttttgcaag |
| OspC | BBB19 | ATGAAAAAGAATACATTAAGTGCATATTTA | AGGTTTTTTTGGACTTTCTGCCAC |
| BmpA | BB0383 | GGATCCAGTGGTAAAGGTAGTCTT | GAATTCTTAAATAAATTCTTTAAGAAACTTCTCAT |
| OspA | BBA15 | ATGAAAAAATATTTATTGGGAATAGGTC | TTTTAAAGCGTTTTTAATTTCATCAAG |

B31 genome designation. Listed are the homologous *B. burgdorferi* B31 genes encoding the proteins based on the genome sequence [1]. The N40 genome is configured differently and some gene sequences vary between N40 and B31.

**Reference**

1. Fraser CM, Casjens S, Huang WM, Sutton GG, Clayton R, et al. (1997) Genomic sequence of a Lyme disease spirochaete, *Borrelia burgdorferi*. Nature 390: 580-586.
